# Emerging Mycotoxins in Cheese: Simultaneous Analysis of Aflatoxin M_1_, Aflatoxicol, and Sterigmatocystin by LC-MS/MS

**DOI:** 10.3390/molecules30081774

**Published:** 2025-04-15

**Authors:** Maurizio Cossu, Andrea Sanna, Giuseppe Mangano, Giuseppe Ledda, Giannina Chessa, Pasquale Gallo, Antonio Vella, Ivan Pecorelli, Stefano Sdogati, Marilena Gili, Carlo Boselli

**Affiliations:** 1 Istituto Zooprofilattico Sperimentale della Sardegna “G.Pegreffi”, Via Vienna, n.2, 07100 Sassari, Italy; maurizio.cossu@izs-sardegna.it (M.C.); andrea.sanna@izs-sardegna.it (A.S.); giuseppe.ledda@izs-sardegna.it (G.L.);; 2 Istituto Zooprofilattico Sperimentale del Mezzogiorno, Via Salute, n.2, 80055 Portici, Italy; pasquale.gallo@izsmportici.it; 3 Istituto Zooprofilattico Sperimentale della Sicilia, Via Gino Marinuzzi, 3, 90129 Palermo, Italy; antonio.vella@izssicilia.it; 4 Istituto Zooprofilattico Sperimentale dell’Umbria e delle Marche, Via G. Salvemini, 1, 06126 Perugia, Italy; i.pecorelli@izsum.it; 5 Istituto Zooprofilattico Sperimentale del Piemonte, Liguria e Valle d’Aosta, Via Bologna, 148, 10154 Torino, Italy; marilena.gili@izsto.it; 6 Istituto Zooprofilattico Sperimentale del Lazio E Della Toscana “M. Aleandri”—National Reference Centre for Ovine and Caprine Milk and Dairy Products Quality (C.Re.L.D.O.C.), 00178 Rome, Italy; carlo.boselli@izslt.it

**Keywords:** emerging mycotoxins, cheese, aflatoxin M_1_, aflatoxicol, sterigmatocystin, LC-MS/MS

## Abstract

The presence of mycotoxins in cheese is a significant concern due to their potential health risks. Mycotoxins can contaminate cheese through two main routes: indirectly via contaminated animal feed, and/or directly, because of mold growth on dairy products. It has been reported that cheese may contain metabolites of aflatoxin B1 such as aflatoxin M1 (AFM1), aflatoxicol (AFL), and, its precursor, sterigmatocystin (STC). This study presents a reliable method for the simultaneous determination of AFM1, AFL, and STC in cheeses made from ovine, goat, or buffalo milk. The method was developed using single liquid extraction, clean-up by an immunoaffinity column (IAC), and liquid chromatography-tandem mass spectrometry (LC-MS/MS) determination. The method was subjected to initial validation according to EU regulations, which outline the required performance parameters and criteria of analytical methods for official food control. The limits of quantification (LOQs) of the method for AFM1, AFL, and STC are 2.0 ng/kg, 5.0 ng/kg, and 1.0 ng/kg, respectively. The method was applied in a study for the assessment of mycotoxin transfer from milk to cheeses and also their growth.

## 1. Introduction

Cheese has been reported to contain mycotoxins, derived from naturally contaminated milk (as raw material) or due to mold growth during cheese spoilage [1,2]. Mycotoxins are toxic secondary metabolites produced by various fungi genera, including *Penicillium, Aspergillus*, and *Fusarium* spp. [3]. The presence of these substances in dairy products can have two origins: (a) indirect contamination, resulting from lactating animals ingesting contaminated feed, and (b) direct contamination, occurring due to accidental mold growth on dairy products [4]. Of major concern is the presence of three toxic mycotoxins: aflatoxin M_1_ (AFM1) and aflatoxicol (AFL), which are metabolites of aflatoxin B_1_ (AFB1), and sterigmatocystin (STC) [3,5]; AFB1 can be usually found at high concentration in contaminated feed materials and, particularly, in maize-based feeds [6,7].

AFM1 is a monohydroxylated derivative of AFB1, formed and excreted in the milk of lactating animals (cattle, buffalo, goat, and sheep) that have consumed AFB1-contaminated products. AFM1 is relatively stable in both raw and processed milk products. Therefore, if raw milk containing AFM1 is used to make cheese, the toxin will be transferred almost quantitatively to cheese [8].

AFL is a reduced metabolite of AFB1, is considered less toxic than AFB1 but easily re-converted into the parent compound by liver dehydrogenases, prolonging the persistence of the mycotoxin in the body [9]. While AFL is soluble in water and easy to dispose of, its formation does not appear to be a significant detoxification pathway for AFB1 [10]. AFL has been found in both artisanal and industrialized cheese from cow milk, at concentrations which are higher if compared to AFM1 found in the same cheese [11].

STC is a fungal secondary metabolite and intermediate in the synthesis of aflatoxins produced by various *Aspergillus* species, with *A. versicolor* being the main producer. *Aspergillus* species that produce STC are generally xerophilic and can grow in environments with low water activity. Optimal environmental conditions for STC production are temperatures between 23 and 29 °C, water activity >0.76, and moisture content above 15%. STC is a biogenic precursor of aflatoxin B1. Despite its potential carcinogenic, mutagenic, and teratogenic properties, STC has been found in different cheeses contaminated with *A. versicolor*. Since 1987, it has been classified by the International Agency for Research on Cancer (IARC) as group 2B, but there is limited information about its occurrence and analysis in food [12,13]. AFM1, AFL and STC structures are detailed in Figure S1.

The European Union (EU) set a maximum concentration level at 0.050 µg/kg for AFM1 in milk while for AFL and STC, no maximum levels are currently in force.

The aim of this study was to develop a suitable and reliable analytical method to support regulatory authorities for future risk-based legislation concerning the maximum levels for mycotoxins in dairy products. The Italian Ministry of Health recently published a document demanding further evaluations for AFL and STC in cheese to fill the knowledge gap about their occurrence in dairy products and also, possibly, determine their transformation factors (TFs) [14].

Analytical procedures based on liquid chromatography coupled to fluorescence detection or mass spectrometry are valuable tools for assessing mycotoxin concentrations in dairy products, notably cheese. In standard analytical methods, an extraction phase utilizes water and organic solvents like acetonitrile and methanol [15,16], or employs innovative systems based on enzyme-assisted extraction with a cocktail of different enzymes, as proposed by Pietri et al. [17]. The subsequent step usually involves a purification phase. Common purification techniques encompass immunoaffinity columns (IACs) [13], Solid-Phase Extraction (SPE) methods [18,19], Molecularly Imprinted Polymers (MIPs) [20,21], and one-step multifunctional clean-up columns (IA Mycosep^®^) [22]. The QuEChERS (Quick, Easy, Cheap, Effective, Rugged, and Safe) method also provides an alternative, covering both extraction and purification steps [23].

For instrumental determination, the standard approach includes reversed-phase high-performance liquid chromatography (HPLC) coupled to a fluorescence detector (FLD) for both AFM1 and AFL [5,24] while UV absorption is typically employed for STC [25]. Liquid chromatography coupled to mass spectrometry (LC-MS) or tandem mass spectrometry (LC-MS/MS) can also be utilized for the determination of mycotoxins in cheese [8]. The analytical multi-mycotoxins methods available in the literature are limited and typically focus on analysis of bovine milk cheese [26].

In this study, we have developed a simple and sensitive method for the simultaneous analysis of AFM1, AFL, and STC, that may occur in different kind of cheeses, made from ovine, caprine and buffalo milk. The analytical approach involved a simple liquid extraction, followed by a clean-up with an immunoaffinity column (IAC); then, the determination was carried out using LC-MS/MS. This method enables the analysis of different cheeses at various stages of ripening, allowing for the quantification of mycotoxins studied at very low levels of contamination; at the present time, only for AFM1 has the Commission Regulation (EU) n. 2023/915 set a maximum limit (ML) in milk and milk-derived products, whereas, regarding AFL and STC, ML are not set yet. The European Food Safety Agency (EFSA) is interested in monitoring AFL and STC levels to evaluate possible health risks for consumers [27].

## 2. Results and Discussion

Firstly, the AFLATEST^®^ WB SR+ immunoaffinity column performances were tested by spiking blank soft, semi-hard, and ripened-hard cheese samples with AFM1, AFL, and STC at 1 ng/g. Recoveries were in the range of 80–100% for AFM1 and AFL, thus proving the suitability of AFLATEST^®^ WB SR+ for AFL determination and overall good preliminary performances for the method. Recoveries for STC were low (<10%), probably due to an unsatisfactory sample clean-up. A freezing step was introduced to precipitate and separate fat and proteins from the liquid extraction phase. Cooling down the sample at −18 °C for 3 h improved the STC recoveries (>70%). A figure describing the improvement in sample clean-up obtained with the freezing step is reported in Supplementary Materials (Figure S2).

The method developed is suitable for AFM1, STC, and AFL analysis in cheese matrixes at very low LOQ values. A comparison between the results obtained in this work and the existing methods in the literature is presented in Table 1. Dairy products are complex matrixes due to their high fat (up to 40%) and protein content, making the analysis of mycotoxins a challenging task. Analytical methods available in the literature are limited and typically focus only on AFM1 analysis in bovine cheese. This study presents an innovative multi-analyte method for the analysis of AFM1, STC, and AFL in cheese obtained from goat, sheep, and buffalo milk (Table 2). The introduction of a purification step using immunoaffinity columns suitable for all three mycotoxins enabled the development of a single-step purification process, effectively eliminating interferences and producing a cleaner final extract. Furthermore, the addition of a freezing step for further purification improved the extract cleanliness and enhanced the efficiency of the immunoaffinity columns. As can be seen, in Table 1, in this work, we obtained comparable recoveries to that reported in the literature by Veršilovskis et al. [19], which appears to have been the best to date. However, examining the precision (RSD_r_), LOD, and LOQ, the present method demonstrates better precision and higher sensitivity compared to what has been reported in the literature so far. These improvements can be addressed by the use of ILIS, compensating for bias and instrumental variability, coupled to IAC purification, which allows for the analysis of only selected compounds, removing all matrix-related interferences from the finial extract. The experiments conducted on cheeses at different ripening stages allowed us to determine the LOQ values, ranging from 1.0 to 5.0 ng/kg (Table 3). The LOQ values comply with the criteria established by Regulation 2782/2023 [28] (0.5 × ML), where ML stands for the “maximum level” reported in Regulation (EU) 915/2023 [27].

**Figure 1:**
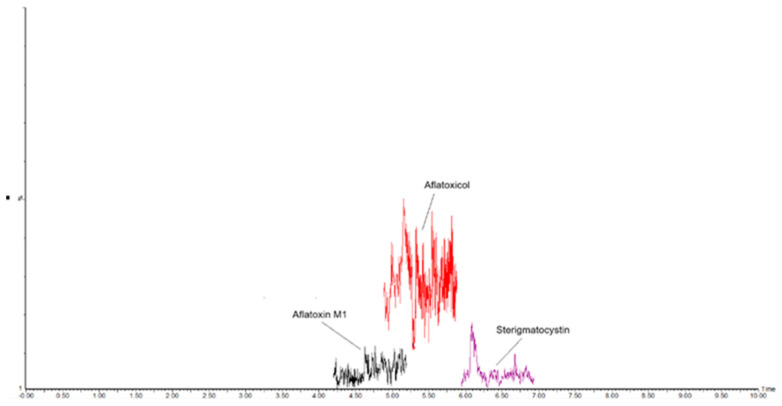
MRM chromatogram of a blank sheep ripened-hard cheese sample (AFM1 < 2.0 ng/kg, STC < 1.0 ng/kg, and AFL < 5.0 ng/kg).

**Figure 2:**
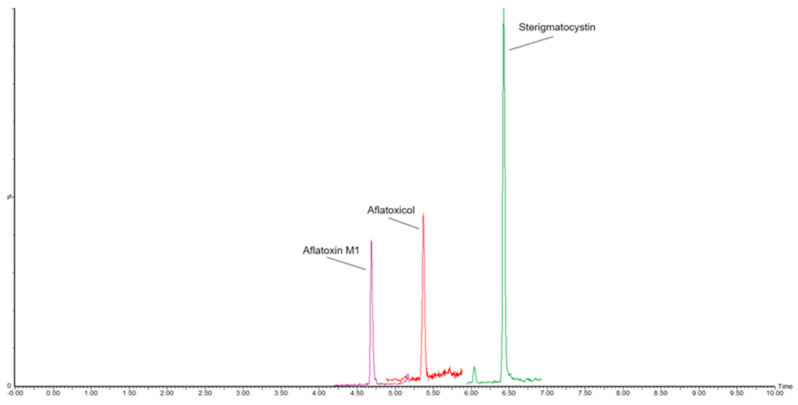
MRM chromatogram of a sheep ripened-hard cheese sample spiked with 25 ng/kg of Aflatoxin M1, 25 ng/kg of Sterigmatocystin, and 50 ng/kg of Aflatoxicol.

**Figure 3:**
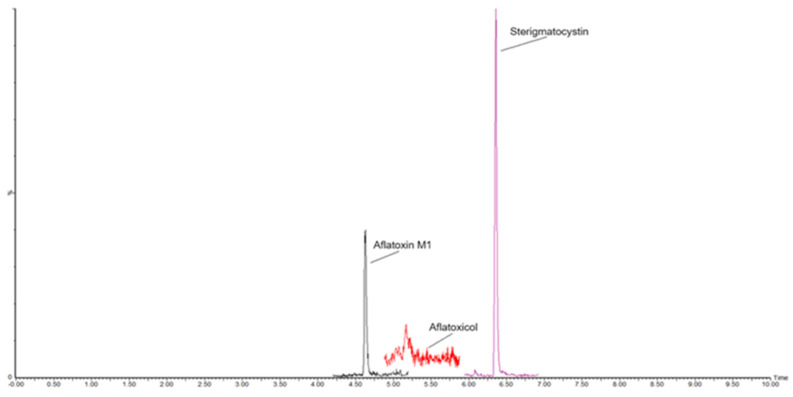
MRM chromatogram of a sheep ripened-hard cheese commercial sample, naturally contaminated with Aflatoxin M1 and Sterigmatocystin at 17 ng/kg and 21 ng/kg, respectively. Aflatoxicol was ≤LOQ.

The RSD_r_ ranged from 2.0 and 4.9% with mean recoveries between 95% and 103%, proving that the developed method complies with performance criteria established by Regulation 2782/2023 for recovery (70–120%) and precision under repeatability conditions (RSD_r_ ≤ 20%). The analytical performances were consistent across different degrees of cheese ripening. Figure 1 illustrates an LC-MS/MS chromatogram of a blank ripened-hard sheep cheese sample; Figure 2 shows the same cheese sample spiked with 25 ng/kg of AFM1 and STC, and 50 ng/kg of AFL; in Figure 3, a chromatogram of a commercial sample of sheep hard cheese, naturally contaminated with AFM1 and STC, is displayed. Supplementary data on precision and accuracy for each cheese type are reported in Table S2.

The high sensitivity and specificity of this method make it particularly useful for food plan control, exposure studies, and risk assessment evaluation, especially for mycotoxins that only recently were put under the magnifying glass of food safety scientists (AFL and STC). The importance of monitoring AFL and STC was recently highlighted by Braun et al., who reported STC in 25% of analyzed processed cereal foods for infants and, for the first time, AFL at a relatively high concentration (1.1 μg/kg) in a milk cereal sample [31]. Cheese is consumed daily by roughly 75% of people between 2 and 19 years in the USA; moreover, cheese consumption is increasing worldwide, with the EU (20.75 kg/year per capita), the USA (17.91 kg/year per capita), and Canada (14.45 kg/year per capita) leading the rankings [32,33]. On these bases, it is evident that, to ensure food salubrity and adequately determine the exposure of populations to contaminants (in this case, mycotoxins), deploying the most sensitive analytical methods available is required.

### Preliminary Results of Cheese Samples Analyzed

This method was applied to the analysis of 55 cheese samples (obtained from sheep, goat, and buffalo milk) with different ripening levels (soft, semi-hard, and ripened-hard cheeses) sampled from local market stores (Sassari, Italy). The method’s high sensitivity allowed for the detection of STC and AFM1 in 95% (52 out of 55) of the cheeses tested, while AFL was not detected in any of the samples (≤5.0 ng/kg). AFM1 was determined at an average level of 11 ng/kg, with concentrations ranging from ≤2.0 to 43 ng/kg, whereas STC was detected at a mean concentration of 5 ng/kg, in a range from ≤1.0 to 24 ng/kg. More precisely, AFM1 was detected in the range 3–37 ng/kg in buffalo cheese, 4–18 ng/kg in goat cheese, and 3–43 ng/kg in sheep cheese; STC was found between 2 and 4.3 ng/kg in buffalo cheese, 2 and 24 ng/kg in goat cheese, and 2 and 17 ng/kg in sheep cheese. Based on cheese ripening, AFM1 was detected in the range 3–37 ng/kg in soft cheeses, 3–18 ng/kg in semi-hard cheese, and 6–43 ng/kg in ripened-hard cheese, while STC was detected at between 2 and 5 ng/kg (soft), 2 and 21 ng/kg (semi-hard), and 3 and 24 ng/kg (ripened-hard). The detected values for each of the 55 analyzed samples are detailed in the Supplementary Materials (Table S3) together with the average levels (Table S4) and were generally lower than the data obtained by the authors, reported in Table 1. From this preliminary market survey, it can be observed how AFM1 cheese contamination is around 10 ng/kg, with no great difference in terms of lactating species or cheese ripening. In contrast, although detected STC levels are generally lower than those of AFM1, they seem to be, on average, higher in ripened than in softer cheese [13]. Possibly, STC and other mycotoxins may be produced by *Aspergillus* spp. and other fungi on the cheese surface during ripening. STC may diffuse under the crust as ripening progresses and thus be detected in these products in higher amounts with respect to soft cheeses, where the ripening stage is reduced or absent.

Referring to AFM1, the detected levels, considering the transformation factors for cheeses, were well below the maximum limit established by the Commission Regulation (EU) n. 2023/915. These preliminary results, obtained for an ongoing study aiming to evaluate the contamination levels of these mycotoxins in cheeses, suggest that both STC and AFM1 can be present while being within safe limits, at least concerning AFM1.

## 3. Materials and Methods

### 3.1. Samples

A total of 11 cheese samples (5 soft, 3 semi-hard and 3 ripened hard) were purchased from local supermarkets. The sampled products included 3 buffalo mozzarella cheeses, 2 buffalo ricotta cheeses, 3 sheep semi-hard cheeses, and 3 sheep hard cheeses. Quality parameters of the cheeses used are reported in Table 2. Cheese hardness parameters were determined using “moisture content on a fat-free basis” (MFFB%) parameter. MFFB% is calculated as [moisture content/(total weight − fat content)] × 100. MFFB was detailed in Council Directive 96/16/EC of 19 March 1996 on statistical surveys of milk and milk products, now repealed by a more recent regulation [34].

### 3.2. Chemicals and Reagents

Reference Material (RM) of Aflatoxin M_1_ was purchased as a solution from Sigma-Aldrich (St. Louis, MO, USA). RMs of Sterigmatocystin, [^13^C_17_]-Aflatoxin M1 and [^13^C_18_]-Sterigmatocystin were purchased as solutions from Romer Labs (Tulln, Austria). RM of Aflatoxicol was obtained as a neat substance from LGC Standards (Wesel, Germany).

Certified Reference Material (CRM) ERM-BD284 was obtained from the EC Joint Research Center (Geel, Belgium).

Acetonitrile (LC-MS-grade) for UHPLC mobile phase and methanol (RPE-grade) for sample extraction were purchased from Carlo Erba (Val-de-Reuil, France). Acetonitrile (RPE-grade) for sample extraction, ammonium acetate (LC-MS-grade) as mobile phase additive, and sodium chloride (RPE-grade) were purchased from VWR (Radnor, PA, USA). Dulbecco’s Phosphate-Buffered Saline (PBS) was obtained from Sigma-Aldrich (St. Louis, MO, USA).

Ultra-pure water used in the preparation of solutions and UHPLC mobile phases was purified by a Milli-Q^®^ Advantage A10 Water Purification System (Merck KGaA, Darmstadt, Germany).

AFLATEST^®^ WB SR+ immunoaffinity columns were obtained from Vicam (Milford, MA, USA). Columns have a specification >90% recovery in complex samples and a capacity as high as 1000 ng for aflatoxins and their metabolites.

### 3.3. Working Standard Solutions

Standards of Aflatoxin M1, Sterigmatocystin, [^13^C_17_]-Aflatoxin M1, and U-[^13^C_18_]-Sterigmatocystin were available as stock solutions. Aflatoxicol stock solution was prepared by dissolution of the neat substance (purity 98%) in methanol at 1000 µg/mL. Aflatoxin M1, Sterigmatocystin, and Aflatoxicol working solutions were prepared at 2.5–2.5–25 ng/mL, respectively, in acetonitrile, starting from stock solutions by successive dilutions. Mixed working solution of labeled Aflatoxin M1 and Sterigmatocystin was prepared at 2.5 ng/mL.

All the solutions were stored in parafilm-coated amber glass bottles in the dark at –18 °C. Appropriate dilutions with water–acetonitrile 75:25 (*v*/*v*) mixture were made to provide standards to be used for calibration purposes.

### 3.4. Sample Preparation and Analysis

An amount of 2.00 ± 0.05 g of homogenized sample were weighed into a centrifuge polypropylene tube and 80 µL of labeled internal standard solution at 2.5 ng/mL was added. The sample was then mixed by an Ultra-Turrax™ (Staufen im Breisgau, Germany) with 2 mL of water and 5 mL of acetonitrile–methanol 85:15 (*v*/*v*) until a milky phase was formed. The extract was centrifuged for 10 min at 9000 rpm and the supernatant was transferred into a 15 mL tube. The remaining residue was extracted again by the above mentioned procedure and the supernatants were combined. The extract was added with 1.0 g of NaCl and stirred vigorously for 1 min. The solubilization of the salt, up to saturation, allows for the separation of the aqueous phase from the organic one. The extract was then placed in a freezer at −18 °C for at least three hours to allow the precipitation of the interfering substances. At the end of this step, 2 mL of organic extract was withdrawn and diluted with 25 mL of PBS. This operation must be carried out immediately after removing the extract from the freezer to avoid re-solubilization of the interferents. The diluted extract was purified through an immunoaffinity column at a flow of about 1 drop/second, avoiding drying out the column. Then, the column was washed by passing 10 mL of water at a rate of about 2 drops/s. At the end of this step, columns were dried by a gentle stream of air for a few seconds.

A glass test tube was placed under the immunoaffinity column and 0.75 mL of the eluting solution of acetronitrile–methanol 1:2 (*v*/*v*) was added and analytes were eluted by gravity. After 3 min, an additional 0.75 mL of the eluting solution was added and eluted by gravity. The column was dried by passing air to quantitatively collect all the eluate. The eluate was dried by a stream of nitrogen at room temperature, taken up again with 0.4 mL of a water–acetonitrile 75:25 (*v*/*v*) mixture with 0.1% formic acid, filtered by 0.2 µm syringe filter, and analyzed by LC-MS/MS.

AFM1 and STC quantification was performed by an isotopically labeled internal standard (ILIS) calibration curve while AFL was quantified by an external standard calibration curve. Concentration levels of the standard calibration curves were between 2.0 and 200 ng/L for AFM1, 1.0–100 ng/L for STC, and 5.0–200 ng/L for AFL. ILIS of AFM1 and STC were at 100 ng/L for each calibration level.

### 3.5. Optimization of the Extraction and Clean-Up Steps

The optimization step that significantly improved the method’s performances primarily targeted the solvent extraction and IA purification. The immunoaffinity columns (IACs) used are certified for isolating aflatoxins B1, B2, G1, G2, M1, M2, and sterigmatocystin, but not aflatoxicol. However, aflatoxicol has a structure very similar to AFM1, allowing it to form an antigen–antibody bond, as noted by the column manufacturers. Preliminary tests were conducted to verify this hypothesis, and experiments at different concentration levels of aflatoxicol confirmed this assumption. The clean-up, particularly the IA purification step, was identified as the most critical phase of the method due to the complexity of the cheese matrix, which contains a wide variety of interferents, including fats and proteins, particularly casein. These components can obstruct the purification columns, thereby limiting the extraction efficiency. To study this effect, recovery tests were carried out using soft, semi-hard, and ripened-hard cheeses, detailed in Table 2, spiked at 1 ng/g. Sample freezing was tested to evaluate extraction efficiency and interference reductions. The cooling process facilitates the mechanical separation of lipid components and proteins, resulting in a cleaner extract and more efficient immunoaffinity purification.

### 3.6. Instrumentation LC-MS/MS

LC-MS/MS analysis was carried out using a Waters XEVO TQ-S™ triple quadrupole instrument equipped with a Waters Acquity UPLC^®^ system with H-Class pumps (Waters Corp., Wilmslow, UK). An electrospray ionization (ESI) probe in positive mode was used. A Waters Acquity UPLC^®^ HSS T3, 1.8 µm (2.1 × 100 mm) column (Waters Corp., Wilmslow, UK) was used. Mobile phases consist of (A) ammonium acetate 1 mM in water and (B) acetonitrile. The gradient was 1 min isocratic at 90% A, then 5 min with a gradient from 90% A to 100% B. Then, 3 min isocratic at 100% B was completed to wash the column and 3 min at 90% A for column re-conditioning and equilibration.

The flow rate was 0.3 mL/min, column temperature was set at 40 °C, and the injection volume was 10 µL.

Under these UHPLC conditions, the AFM1, AFL, and STC retention times were 4.7, 5.4, and 6.4 min, respectively. The parameters of the mass spectrometer were optimized using the standard solution of each analyte by combined infusion.

The best response was recorded with the following parameters: source temperature 150 °C, capillary voltage 2.0 kV, nitrogen desolvation gas temperature 600 °C with a flow rate of 1000 L/h, cone gas flow 150 L/h, and nebulizer 5 bar. Each analyte of interest was surveyed for optimal conditions and monitored by multiple reaction monitoring (MRM). MS transitions and cone and collision parameters are listed in Table S1 (Supplementary Materials).

### 3.7. Method Performance Parameters

The study of method performances was conducted in accordance with Regulation (UE) 2017/625 [35] and Regulation (EU) n. 2023/2782 [28], which outlines the required parameters and criteria for analytical methods used in the official control of mycotoxins in food. Specifically, the study included the following parameters: linearity and working range, limit of detection (LOD), limit of quantitation (LOQ), accuracy (in terms of extraction recovery), precision (in terms of relative standard deviation in repeatability conditions − RSD_r_), and specificity. Data for all these parameters were obtained using spiked samples or Certified Reference Materials when available.

As linearity criteria were not pointed out by [28,35] this parameter was evaluated based on Guidance SANTE 11312/2021v2 [36]. Linearity was evaluated in solvent using calibration standard curves at different concentration levels for each mycotoxin. The acceptability criteria is a ± 20% tolerance window of back-calculated concentrations (BCCs) of each calibration point from the true value. The concentration ranges studied for the different mycotoxins are presented in Table 3. The instrumental LOD (limit of detection) was evaluated as the concentration showing a signal-to-noise (S/N) ratio ≥ 3 using solvent solutions, while the instrumental LOQ (limit of quantitation) was estimated as the lowest concentration of the analyte in the sample that could be measured with reasonable statistical certainty (S/N ≥ 10). The LOQ values obtained for the three mycotoxins are reported in Table 3.

Accuracy and precision were evaluated using spiked blank cheese samples at 25 ng/kg for AFM1 and STC and 50 ng/kg for AFL (Level 1); 50 ng/kg for AFM1 and STC, 500 ng/kg for AFL (Level 2) and reported in Table 4.. Additionally, accuracy for AFM1 was assessed analyzing a Certified Reference Material milk powder matrix for AFM1 (ERM-BD 284) (Table 5).

The specificity of the method was evaluated through an analysis of different kinds of blank and spiked samples. Specificity criteria were met if the spiked sample signal was at least three times higher than interferences, if any, present in a blank sample [28]. The applicability was estimated through the comparison of retention times (RTs) and MS/MS parameters such as the ion ratio% (IR), which is the ratio between the two MRM transitions of each molecule (qualifier/quantifier q/Q × 100). Mycotoxins were identified in an unknown sample if the IR was between ±30% of that of the standard. Regarding the RT, for AFM1 and STC, the relative retention time (RRT) was used, while for AFL, the absolute retention time was considered. In the first case, an identification range window of ±0.02 min was used, while for AFL, a range of ±0.1 min around the RT of the reference standard was considered. Furthermore, samples of uncontaminated cheeses were used to verify the absence of matrix interference in the MS/MS chromatograms.

### 3.8. Statistical Analysis

The descriptive parameters of the calibration curve (equation, correlation coefficient, and back-calculated concentrations) were calculated using the instrumental software TargetLynx V4.1 (Waters™, Milford, MA, USA) with the linear regression model. Extraction recovery was calculated as the ratio% between experimental sample concentration (calculated via calibration curve equation) and spiked concentration.

Precision was assessed as the relative standard deviation in repeatability conditions (RSD_r_). Both precision and accuracy were calculated using Microsoft Excel (Microsoft Excel 2007, Microsoft Corporation, version 2501, Redmond, WA, USA).

## 4. Conclusions

A method combining Ultra-Turrax™ sample grinding in the presence of a solvent, followed by clean-up with an immunoaffinity column and LC-MS/MS determination in positive MRM mode, has been developed and optimized. The introduction of a freezing step facilitated the separation of lipid components and proteins that could clog the immunoaffinity column. This additional step increased its efficiency in terms of recovery.

The analytical performance parameters were evaluated for the quantification of Aflatoxin M1, Aflatoxicol, and Sterigmatocystin in goat, sheep, and buffalo cheeses, at level concentrations fitting for the purposes of food control. The optimization approach allowed for the development of a quantitative method, with remarkable sensitivity, excellent linearity response, and adequate precision for the mycotoxins studied, which can also be applicable to exposure evaluations. Only a few methods in the literature are available to quantify AFL and STC and, to the best of our knowledge, the proposed method is the most sensitive. In comparison, this method offers several advantages, including applicability to a wide range of cheeses and suitability for routine analysis. The preliminary results obtained for an ongoing study aimed at evaluating the contamination levels of these mycotoxins in cheeses, demonstrating the applicability of this method.

## Figures and Tables

**Table 1:** Comparison between the present and previously published methods.

		Recovery %	RSD_r_ %	LOD (ng/kg)	LOQ (ng/kg)	Approximate RT (min)
Pietri et al., 2016 [17]	AFM1	87.1–96.3	1.4–2.3	5–20	15–60	4.9
	STC	-	-	-	-	-
	AFL	-	-	-	-	-
Iha et al., 2011 [15]	AFM1	80	5.9	3	10	7.0
	STC	-	-	-	-	-
	AFL	-	-	-	-	-
Rodríguez-Cañàs et al., 2023 [29]	AFM1	79.6–101.1	6.63–13.20	6–11	20–36 *	5.9
	STC	73.9–104.3	1.03–5.25	178–1005	588–3316 *	8.4
	AFL	-	-	-	-	-
Carvajal-Moreno et al., 2019 [5]	AFM1	95	-	10	-	8.5–9.5
	STC	-	-	-	-	-
	AFL	98	-	10	-	3.0–5.6
Jakšić et al., 2021 [30]	AFM1	84–112	8–18	10	30	5.7
	STC	-	-	-	-	-
	AFL	-	-	-	-	-
Veršilovskis et al., 2009 [19]	AFM1	-	-	-	-	-
	STC	96–104	17–27	30	100	7.7
	AFL	-	-	-	-	-
Current Study(This work)	AFM1	95	2.0–3.2	0.6	2.0	4.7
	STC	98–103	2.1–2.7	0.2	1.0	6.4
	AFL	96–97	2.7–4.9	1.0	5.0	5.4

*: in concentrated extracts.

**Table 2:** Quality parameters of cheeses used for study method.

Composition Parameter	Soft (MFFB% ≥ 62)	Semi-Hard (62 > MFFB% > 55)	Ripened-Hard (MFFB% ≤ 55)
Protein %	15–19	29	29–34
Fat %	20–40	40	31–34
Dry matter %	>30	>70	>75

**Table 3:** Linearity range, LOD, and LOQ.

Mycotoxin	Linearity Range (ng/kg)	Calibration Curve Equation	R^2^	LOD (ng/kg)	LOQ (ng/kg)
AFM_1_	2.0–200	y = 2.05x − 0.38	0.9986	0.6	2.0
STC	1.0–100	y = 1.26x − 0.15	0.9999	0.2	1.0
AFL	5.0–2000	y = 136.4x − 135.9 *	0.9982	1.0	5.0

* External standard calibration.

**Table 4:** Method performance evaluation.

Mycotoxin	Replicates (*n*)	Concentration Range (ng/kg)	Performance Evaluation Parameters	
			Recovery %	RSD_r_ %
AFM_1_	6	25	95	2.0
	5	50	95	3.2
STC	6	25	103	2.1
	5	50	98	2.7
AFL	5	50	97	2.7
	5	500	96	4.9

RSDr: relative standard deviation under repeatability conditions.

**Table 5:** Method performance evaluation on CRM ERM-BD 284.

Certified Reference Material	AFM1 Concentration (ng/kg)	Replicates *(n)*	Recovery %	RSD_r_ %
ERM BD 284—Milk powder	440	10	101	3.6

## Data Availability

The original contributions presented in this study are included in this article and the Supplementary Materials. Further inquiries can be directed to the corresponding authors.

## Supplementary Materials

The following supporting information can be downloaded at https://www.mdpi.com/article/10.3390/molecules30081774/s1: Figure S1: Structures of mycotoxins examined in cheese samples. Figure S2: Sample clean-up without freezing step (left) and with freezing step (−18 °C, 3 h) (right). Table S1. MS transitions and cone and collision parameters. Table S2. Recovery and precision performances at two concentration levels for the different kinds of cheese. Table S3. AFM1, AFL, and STC contamination of 55 cheese samples. Table S4. Average AFM1 and STC concentrations in the analyzed samples.

## Abbreviations

The following abbreviations are used in this manuscript:

AFL	Aflatoxicol
AFB1	Aflatoxin B1
AFM1	Aflatoxin M1
BCC	Back-calculated concentrations
CRM	Certified Reference Material
EFSA	European Food Safety Agency
EU	European Union
ILIS	Isotopically labeled internal standard
HPLC	High-performance liquid chromatography
IAC	Immunoaffinity column
IR	Ion ratio
LC-MS/MS	Liquid chromatography-tandem mass spectrometry
LOD	Limit of detection
LOQ	Limit of quantitation
MIP	Molecularly Imprinted Polymers
MRM	Multiple reaction monitoring
PBS	Phosphate-Buffered Saline
RM	Reference Material
RSDr	Relative standard deviation under repeatability conditions
RT	Retention time
SPE	Solid-Phase Extraction
STC	Sterigmatocystin
