# Supplementary material for: Emerging Mycotoxins in Cheese: Simultaneous Analysis of Aflatoxin M1, Aflatoxicol, and Sterigmatocystin by LC-MS/MS
Source: Molecules. 2025 Apr 15;30(8):1774. doi: 10.3390/molecules30081774 (PMC12029257; doi:10.3390/molecules30081774)

Supplementary materials

Table S1. MS transitions, cone and collision parameters.

Mycotoxin	Precursor ion (m/z)	Molecular ion	Cone voltage (V)	Quant (Q) (m/z)	Qual 1 (q) (m/z)	Qual 2 (q2) (m/z)	CE (Q) (eV)	CE (q) (eV)	CE (q2) (eV)
AFM_1	329	$[M+H]^+$	20	273	259	229	22	23	38
AFL	297	$[M-H_2O+H]^+$	50	269	141	115	20	48	55
STC	325	$[M+H]^+$	30	310	281	253	24	35	42
$^{13}C_{17}$-AFM_1 (IS)	346	$[M+H]^+$	20	288	242	-	22	38	-
$^{13}C_{18}$-STC (IS)	343	$[M+H]^+$	30	327	297	-	24	35	-

$AFM_{1:}$ Aflatoxin M1; AFL: Aflatoxicol; STC: Sterigmatocystin; $^{13}C_{17}$-AFM_1: $^{13}C_{17}$ Aflatoxin M1; $^{13}C_{18}$-STC: $^{13}C_{18}$ Sterigmatocystin; CE: Collision energy

Table S2. Recovery and precision performances at two concentration levels for the different kind of cheese.

		**Soft Cheese**				**Semi-hard Cheese**				**Ripened-hard cheese**		
	**n.**	**Spiked conc. (ng/kg)**	**Rec. %**	**RSD_r %**	**n.**	**Spiked conc. (ng/kg)**	**Rec. %**	**RSD_r %**	**n.**	**Spiked conc. (ng/kg)**	**Rec. %**	**RSD_r %**
AFM1	5	50	95	3.2	3	25	97	1.4	3	25	94	2.6
STC	5	50	98	2.7	3	25	105	2.7	3	25	102	1.5
AFL	5	500	96	4.9	3	50	99	3.4	3	50	96	2.0

$AFM_{1:}$ Aflatoxin M1; AFL: Aflatoxicol; STC: Sterigmatocystin: Rec%: Recovery; RSDr: relative standard deviation under repeatability conditions

Table S3. AFM1, AFL and STC contamination of 55 cheese samples.

Sample	Species	Cheese type	AFM1 (ng/kg)	STC (ng/kg)	AFL (ng/kg)
1	Buffalo	Soft	4	2	< LOQ
2	Goat	Soft	10	4	< LOQ
3	Goat	Ripened-Hard	4	9	< LOQ
4	Sheep	Ripened-Hard	7	3	< LOQ
5	Sheep	Ripened-Hard	13	6	< LOQ
6	Sheep	Ripened-Hard	16	16	< LOQ
7	Sheep	Semi-Hard	16	17	< LOQ
8	Sheep	Soft	10	2	< LOQ
9	Sheep	Ripened-Hard	3	6	< LOQ
10	Sheep	Ripened-Hard	7	7	< LOQ
11	Sheep	Semi-Hard	12	9	< LOQ
12	Goat	Ripened-Hard	17	21	< LOQ
13	Sheep	Soft	9	2	< LOQ
14	Sheep	Ripened-Hard	11	6	< LOQ
15	Sheep	Ripened-Hard	18	8	< LOQ
16	Buffalo	Soft	5	< LOQ	< LOQ
17	Buffalo	Soft	5	2	< LOQ
18	Sheep	Semi-Hard	9	5	< LOQ

19	Sheep	Ripened-Hard	13	4	< LOQ
20	Sheep	Semi-Hard	43	3	< LOQ
21	Goat	Semi-Hard	15	24	< LOQ
22	Buffalo	Soft	7	3	< LOQ
23	Buffalo	Soft	6	3	< LOQ
24	Sheep	Soft	< LOQ	< LOQ	< LOQ
25	Goat	Soft	< LOQ	< LOQ	< LOQ
26	Buffalo	Soft	37	4	< LOQ
27	Goat	Soft	7	2	< LOQ
28	Buffalo	Soft	5	2	< LOQ
29	Buffalo	Soft	6	< LOQ	< LOQ
30	Buffalo	Soft	25	< LOQ	< LOQ
31	Buffalo	Soft	3	< LOQ	< LOQ
32	Buffalo	Soft	5	3	< LOQ
33	Buffalo	Soft	27	3	< LOQ
34	Sheep	Ripened-Hard	4	2	< LOQ
35	Sheep	Semi-Hard	12	6	< LOQ
36	Sheep	Semi-Hard	17	3	< LOQ
37	Sheep	Ripened-Hard	5	10	< LOQ
38	Sheep	Semi-Hard	14	16	< LOQ
39	Sheep	Semi-Hard	6	6	< LOQ
40	Goat	Soft	18	4	< LOQ
41	Goat	Soft	15	5	< LOQ
42	Goat	Soft	11	4	< LOQ
43	Sheep	Soft	< LOQ	<LOQ	< LOQ
44	Sheep	Soft	4	2	< LOQ
45	Buffalo	Soft	5	2	< LOQ
46	Buffalo	Soft	17	2	< LOQ
47	Buffalo	Soft	11	2	< LOQ
48	Buffalo	Soft	27	3	< LOQ
49	Buffalo	Soft	5	2	< LOQ
50	Sheep	Semi-Hard	6	6	< LOQ
51	Buffalo	Soft	8	2	< LOQ
52	Buffalo	Soft	8	2	< LOQ
53	Sheep	Semi-Hard	9	6	< LOQ
54	Buffalo	Soft	4	< LOQ	< LOQ
55	Buffalo	Soft	7	2	< LOQ

Table S4 . Average AFM1 and STC concentrations in the analyzed samples.

**Species**		
	**AFM1 (ng/kg)**	**STC (ng/kg)**
Buffalo	11	2
Goat	12	9
Sheep	11	7
**Type of cheese**		

Soft	11	3
Semi-Hard	14	9
Ripened-Hard	10	8

Figure S1. Structures of mycotoxins examinated in cheese samples.

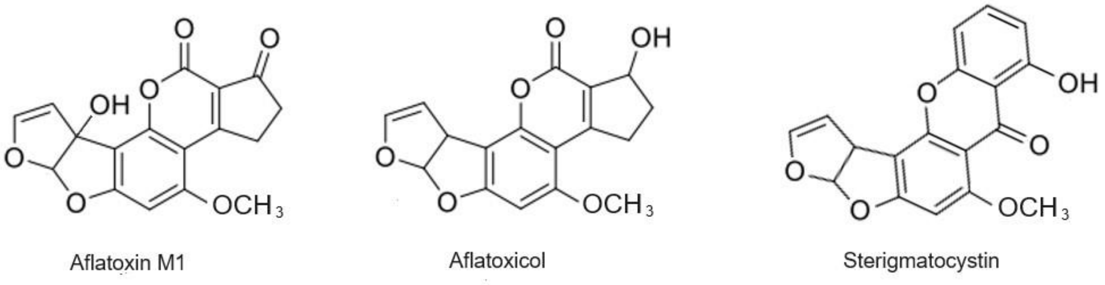

Figure S2. Sample clean-up without freezing step (left) and with freezing step (-18°C, 3 h) (right)

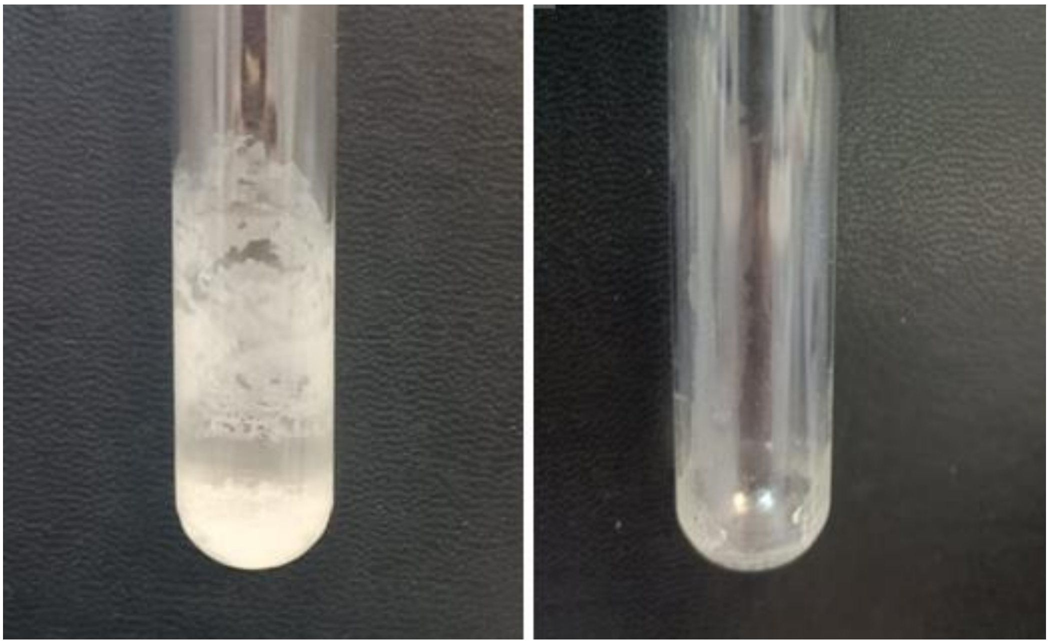

Supplement: Supplementary file 1 [file molecules-30-01774-s001.zip › molecules-3574798-supplementary.pdf]
